# Elective Total Joint Replacement in Patients With Left Ventricular Assist Devices: A Modern Case Series and Systematic Review

**DOI:** 10.1177/15563316261464135

**Published:** 2026-07-06

**Authors:** Ramana Kolady, Natasha Singh, Heather L. Lander, Benjamin F. Ricciardi

**Affiliations:** 1 Department of Orthopaedic Surgery, University of Rochester School of Medicine, NY, USA; 2 Department of Anesthesiology, University of Rochester School of Medicine, NY, USA; 3 Center for Musculoskeletal Research, University of Rochester School of Medicine, NY, USA

**Keywords:** left ventricular assist device, total hip arthroplasty, total knee arthroplasty, complications

## Abstract

**Background:**

Advances in technology for left ventricular assist devices (LVADs) have improved their performance and increased their use as a destination therapy for advanced heart failure. As patients with heart failure live longer, there is a need to develop coordinated care models to offer elective musculoskeletal surgeries for these patients.

**Purpose:**

We sought to (1) determine postoperative outcomes in patients with LVADs undergoing elective total joint replacement (TJR) in the setting of multidisciplinary protocol-driven perioperative testing and care coordination and (2) perform a systematic review of the literature regarding the outcomes and complications of elective surgeries in patients with LVADs.

**Methods:**

We conducted a retrospective, single-center case series of patients with LVADs who underwent elective hip, knee, or shoulder arthroplasty between January 2020 and July 2024. Only patients with an LVAD at the time of their elective surgery were included (5 patients who underwent 6 surgeries). All patients were managed according to a standardized, multidisciplinary protocol and had a minimum of 1 year of clinical follow-up. The primary outcomes were in-hospital and postoperative complications, readmissions, and reoperations. We also performed a systematic review examining the outcomes of elective surgeries on patients with LVADs. We used the search terms “left ventricular assist device,” “elective surgery,” and “LVAD and arthroplasty.” From the 18 studies included in the final analysis, we extracted data on study design, level of evidence, sample size, type of elective surgery, duration of follow-up, reoperations, and complications.

**Results:**

In the 5 patients with LVADs who underwent 6 TJR procedures, the most common complication was perioperative bleeding requiring transfusion. There were no reoperations over a 22-month average follow-up period. The systematic review showed higher rates of perioperative mortality, reoperation, and infection compared to our case series with more modern LVADs.

**Conclusion:**

The results of this retrospective, single-institution case series and systematic review suggest that TJR may be considered in patients with modern LVADs, provided that robust perioperative management and multidisciplinary support are in place. While anticoagulation and bleeding risk remain important considerations, improvements in device design and perioperative management have substantially mitigated these concerns.

**Level of Evidence:**

Level V: retrospective case series and systematic review of Level II to Level V studies.

## Introduction

Left ventricular assist devices (LVADs) have changed the management of patients with advanced heart failure, serving as both a bridge to transplant and as a destination therapy for patients who are ineligible for transplantation. Advances in LVADs have demonstrated improved hemodynamic performance, durability, and patient survival.^1-3^ As patients with LVADs live longer, they increasingly face age-related comorbidities that may require elective surgeries to maintain function, mobility, and quality of life. While existing literature on patients with LVADs has examined elective cardiac procedures, there is little research on elective noncardiac surgeries, specifically those involving the musculoskeletal system, which are among the most common elective surgeries in the United States.^4^ It is important to explore the unique perioperative planning, optimization, and management of this patient population.

Total joint replacement (TJR) is one of the most common elective procedures performed in the United States, with more than 1 000 000 total hip and knee replacements occurring annually.^4^ The volume of these procedures is expected to rise with the growing prevalence of arthritis and the number of people seeking improved mobility and quality of life.^5,6^ However, very few studies analyzing TJR outcomes in patients with LVAD devices have been conducted, and most of them are older, without the use of more modern advanced recovery protocols.^7,8^ Issues that are unique to this patient population in the setting of TJR include bleeding events secondary to anticoagulation, thrombotic events, need for postoperative transfusion, and stroke.^7-10^ For example, one 2022 study of 5 patients who had 12 surgeries found all LVAD-implanted total knee arthroplasty (TKA) patients experienced thrombotic events; 8 of 12 surgeries required an intraoperative transfusion.^8^ Moreover, they found that 2 out of 5 patients who underwent revision TKA for prosthetic joint infection passed away secondary to hemorrhagic stroke at 16 and 20 months following revision surgery.

With advancements in LVAD technology and its increasing use as a destination therapy for patients with advanced heart failure, there is a need for coordinated care models for offering elective musculoskeletal surgeries to improve quality of life, mobility, and function. We aim to (1) present postoperative outcomes including complications, readmissions, and perioperative mortality rates in patients with LVADs undergoing elective TJR in the setting of a single institution’s multidisciplinary protocol and (2) perform a systematic review of the literature regarding the outcomes and complications of elective surgeries in patients with LVADs. An improved understanding of TJR in this cohort may inform and advance evidence-based guidelines for surgical risk assessment, perioperative planning, and postoperative care efforts.

## Methods

### Retrospective Case Series

We performed a retrospective, single-center case series of patients with LVADs who underwent elective hip, knee, or shoulder arthroplasty between January 2020 and July 2024. Only patients with an LVAD at the time of their elective surgery were eligible for inclusion (n = 5 patients underwent 6 surgeries; Table 1). All patients had a minimum 1-year of clinical follow-up. No patients were excluded from the study. Three patients underwent a total of 4 total hip arthroplasty (THA) procedures, 1 patient underwent TKA, and 1 patient underwent a total shoulder arthroplasty (TSA) procedure. Patient medical records were examined for details on age, sex, comorbidities, LVAD duration, LVAD model, preoperative anticoagulation, and preoperative optimization measures undertaken. Characteristics of their surgeries were recorded, including cement usage, intraoperative blood loss, need for transfusion, and disposition from the operating room. Primary outcomes were in-hospital and postoperative complications, readmissions, and reoperations over the follow-up period. This study was approved by our Institutional Review Board.

**Table 1.** Preoperative Details of LVAD Patients Undergoing THA/TKA/TSA Procedures.

Age	Sex	Procedure	Cemented or cementless	LVAD duration	LVAD model	Preoperative anticoagulation	Preoperative optimization
62	Male	THA	Cementless	3.53 y	HM3	Warfarin and Plavix (held prior to procedure, resumed postoperatively)	RHC with Swan placement and IV heparin 2 d prior to surgery (stopped morning of surgery). ECHO completed 4 mo prior to surgery
62	Male	THA	Cementless	1.56 y	HM3	Warfarin and ASA (held prior to procedure, resumed postoperatively with Lovenox)	RHC with LVAD ramp speed optimization ECHO 1 wk prior to procedure. Femoral and lateral femoral cutaneous nerve blocks administered day of operation
		THA	Cementless	1.15 y	HM3	Coumadin (held for 5 d prior to procedure, resumed postoperatively), ASA resumed postoperatively	Femoral nerve block applied day of operation. Had cardiac catheterization 3 mo prior to surgery. Most recent ECHO completed 10 mo prior to operation
74	Male	THA	Cemented	10.31 y	HM2	Not on anticoagulation, was on coumadin (stopped due to gastric bleeding)	ECHO 2 wk prior to procedure, RHC with leave in Swan day of procedure
62	Male	TKA	Cemented	2.4 y	HM3	Warfarin, Aspirin (held 5 d prior to procedure, resumed postoperatively with Lovenox)	Adductor canal nerve block day of procedure. Catheterization completed 3 mo prior to procedure. Last LVAD with ECHO completed 7 mo prior to procedure
73	Male	TSA	Cementless	10.91 y	HM2	Warfarin (resumed postoperatively with Lovenox), ASA (held prior to and post-surgery)	RHC with leave in Swan and IV heparin day prior to procedure. Received interscalene nerve block day of procedure. Received ECHO 2 mo prior to procedure

Abbreviations: ASA, American Society for Anesthesiologists; ECHO, echo cardiogram; LVADs, left ventricular assist devices; RHC, right heart catheterization; THA, total hip arthroplasty; TKA, total knee arthroplasty; TSA, total shoulder arthroplasty.

All patients with implanted LVADs who underwent elective TJR at our institution were managed according to a standardized, multidisciplinary protocol established by the cardiac anesthesiology division and heart failure service. Preoperative assessment included a Center for Perioperative Medicine evaluation that involved comprehensive cardiac optimization and device evaluation, specifically consisting of LVAD interrogation with ramp echocardiography within 3 months, right heart catheterization within 6 months, and pacemaker/implantable cardioverter-defibrillator interrogation within 3 months of surgery. Laboratory testing, pulmonary function studies, and imaging were obtained per institutional policy. Disease states optimized included diabetes, hypertension, smoking cessation, anemia, and vulnerability (based on the FRAIL scale, nutrition, cognition, etc). Candidates had to demonstrate stable hemodynamics, LVAD parameters, and medical comorbidities prior to surgical clearance. A multidisciplinary conference comprising representatives from orthopedic surgery, cardiac anesthesiology, heart failure cardiology, cardiac intensive care unit (ICU), cardiac surgery, perfusion, nursing, and rehabilitation services was held within 2 weeks of the planned operation to review patient status, define intraoperative hemodynamic targets, and establish contingency plans for right ventricular failure or mechanical circulatory support.

Elective joint procedures were scheduled as first-start cases in large operating suites to accommodate specialized monitoring and personnel. Anesthetic management was performed or supervised by a cardiac anesthesiologist, with perfusion and VAD-trained staff present throughout. Standard American Society for Anesthesiologists monitors were supplemented by invasive arterial and central venous access; transesophageal echocardiography was available for intraoperative assessment as indicated. General anesthesia with endotracheal intubation was the preferred technique, with hemodynamic goals targeting a mean arterial pressure of 70 to 80 mmHg and meticulous volume management to preserve right ventricular function. Anticoagulation was managed in consultation with the heart failure and surgical teams according to LVAD type (HeartMate II vs HeartMate 3) and institutional bridging protocols. Postoperatively, patients recovered in either the cardiac ICU or the post-anesthesia care unit (PACU), depending on the complexity of the procedure and extubation status. Early initiation of physical therapy, pulmonary hygiene, and LVAD-handling education was emphasized to facilitate recovery and minimize perioperative complications.

### Systematic Review

A systematic review of the literature was performed using the PubMed database to identify all relevant literature according to preferred reporting items for systematic reviews and meta-analyses (PRISMA) guidelines. We used the search terms “left ventricular assist device,” “elective surgery,” and “LVAD and arthroplasty.” No additional queries or keywords were employed in any combination. All full-text studies in English detailing outcomes of patients with LVADs in the setting of noncardiac elective surgeries were included for data extraction. We excluded all studies that were not written in English, did not have full-text availability, did not involve long-term LVADs, focused on non-elective surgeries, or focused on elective, cardiac procedures. Extracted data included information on study design, level of evidence, sample size, type of elective surgery, duration of follow-up, reoperations, and complications.

## Results

The average length of stay across patients at our institution was 4.8 days (range 4–7 days; Table 2). In 4 cases, patients were sent to the PACU after the surgical procedure and were then discharged to a regular floor. In 2 cases, both THAs, the patients were transferred to the cardiac ICU after surgery for closer monitoring, subsequently to a regular floor, after which they were discharged. Two patients received a 1 unit transfusion of packed red blood cells during their stay. Three surgeries did not have postoperative complications or readmissions. One patient developed worsening hypoxia during the hospital stay requiring cardiac ICU admission for diuresis; this resolved without further issues. One patient had a hospital readmission 9 days after surgery due to hypotension and gastrointestinal (GI) bleed; this patient had a history of GI bleeding with aspirin usage. The average follow-up time was up to 22 months. No patients underwent reoperations. One patient passed away 1.2 years after TJR and 11.6 years after LVAD implantation due to ongoing issues with melena and fluid overload.

**Table 2.** Postoperative Details of LVAD Patients Undergoing THA/TKA/TSA Procedures.

Procedure	Disposition after OR	LOS	Transfusion	Complications	Reoperation	Follow-up	Mortality
THA	PACU	7 d	None	Nerve pain early post-procedure after fall from bed; no other unexpected complications	No	39 mo	Not deceased; underwent heart transplant subsequently
THA (side 1)	PACU	4 d	None	No unexpected complications	No	21 mo	Not deceased
THA (side 2)	CICU	4 d	1 Unit PRBC	Worsening hypoxia; transferred PACU to CICU, later to floor; no other complications	No	15 mo	Not deceased
THA	CICU	5 d	1 Unit PRBC	Hypotension; readmission 9 d after procedure, felt weak, had melena. Admitted to floor for gastric bleeding (history of anticoagulant use)	No	15 mo	Deceased at 15 mo post-surgery secondary to gastric bleeding and hospitalizations
TKA	PACU	4 d	None	No unexpected complications	No	22 mo	Not deceased
TSA	PACU	5 d	None	No unexpected complications	No	9 mo	Not deceased

Abbreviations: CICU, cardiac intensive care unit; LOS, length of stay; LVADs, left ventricular assist devices; OR, operating room; PACU, post-anesthesia care unit; PRBC, packed red blood cells; THA, total hip arthroplasty; TKA, total knee arthroplasty; TSA, total shoulder arthroplasty.

**Figure 1.**
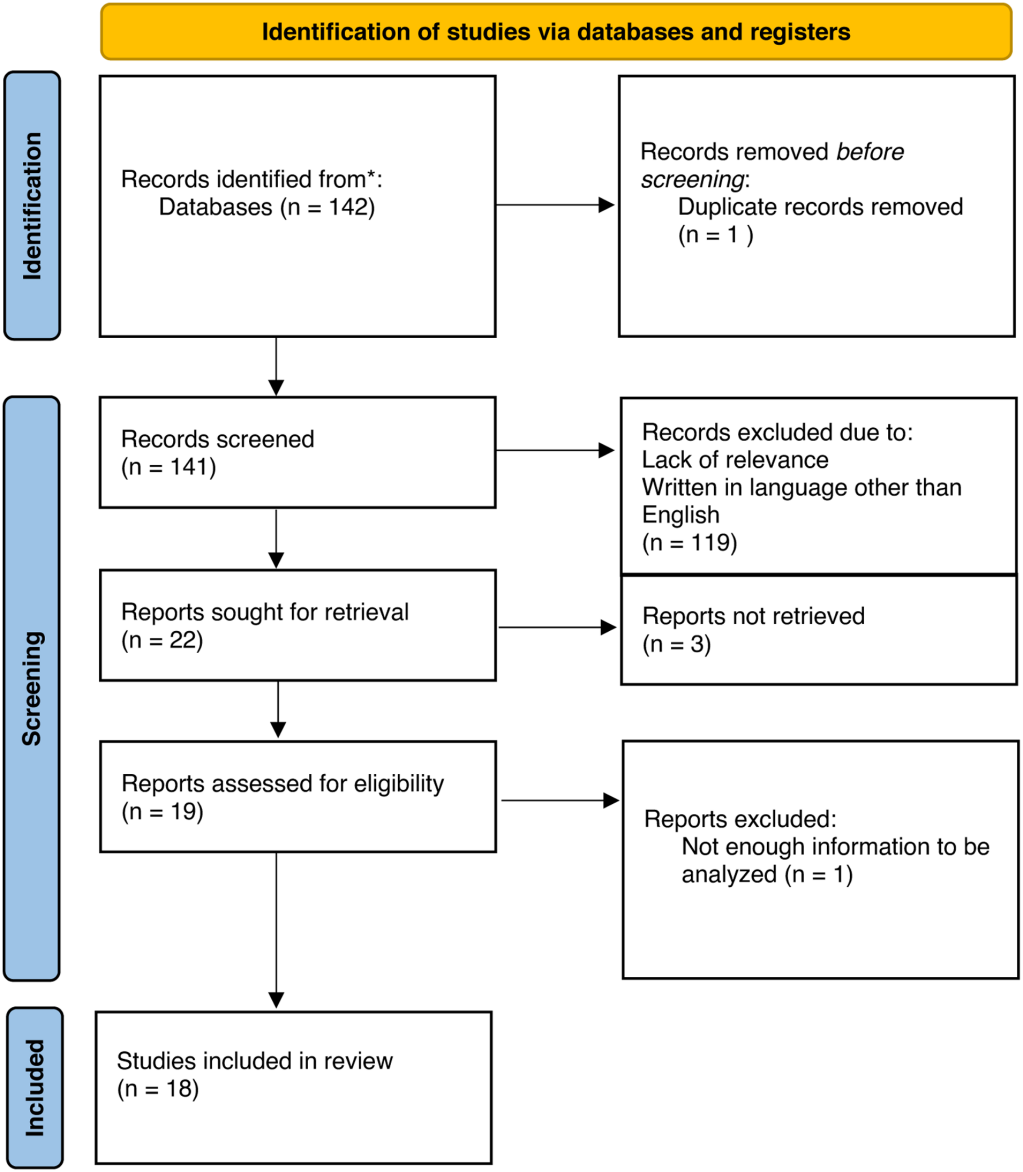
PRISMA flow diagram.

For the systematic review, the initial search yielded 142 articles. After removal of 1 duplicate study, 2 independent reviewers evaluated all studies according to inclusion and exclusion criteria; 119 studies were excluded. Twenty-two studies met criteria for inclusion and were examined for data extraction; 3 studies could not be accessed and were excluded. A fourth study did not have enough information reported to be analyzed, leaving 18 studies in the final analysis (Figure 1).

The systematic review found only 2 studies detailing outcomes in patients with implanted LVADs who underwent elective musculoskeletal surgeries (Table 3 and Supplemental Table 1). One study found a higher rate of perioperative complications than in our case series; they reported a 67% transfusion rate and all patients experienced either a thrombotic event or a bleeding event requiring transfusion.^8^ Non-orthopedic surgery publications report varying rates of postoperative complications depending on the procedure performed and the acuity of the surgery. For instance, Yahav-Shafir et al reported a 9% mortality rate and a 39% transfusion rate after a mixture of elective and emergent general surgery procedures.^9^ Mentias et al reported a 4% 30-day mortality rate, 25% acute kidney injury rate, and 24% blood transfusion rate in more urgent/emergency general surgery cases in patients with LVADs.^11^ Blood transfusion rates were also high in reports of more elective than emergent general surgery procedures, with Morgan et al reporting a 36% blood transfusion rate.^12^ Emergent general surgery cases appeared to fare worse, with 58% having complications after surgery including a 17% rate of sepsis.^13^

**Table 3.** Characteristics of Included Studies.

Authors	Year	Title	Level of evidence	Study design	Sample size
Sheikh et al^7^	2025	Primary neuraxial anesthetic for elective total knee arthroplasty in patient with left ventricular assist device	Level V	Case report	1
Rosenberg et al^8^	2022	Total joint arthroplasty in patients with an implanted left ventricular assist device	Level IV	Retrospective case series	5
Stevenson et al^22^	2025	Temporarily reversing warfarin with low-dose 4-factor prothrombin complex concentrate in left ventricular assist device patients undergoing an invasive procedure	Level III	Retrospective cohort study	14
Yahav-Shafir et al^9^	2024	Quality improvement in anesthetic management of patients with left ventricular assist device support presenting for noncardiac surgery: A single-center experience	Level III	Retrospective cohort study	23
Mentias et al^11^	2020	Trends, perioperative adverse events, and survival of patients with left ventricular assist devices undergoing noncardiac surgery	Level III	Retrospective cohort study	8118
Zilbermints et al^23^	2020	Abdominal surgery in patients with a ventricular assist device: A single-center experience in Israel	Level III	Retrospective cohort study	93
Vigneswaran et al^24^	2019	Laparoscopic procedures in patients with cardiac ventricular assist devices	Level IV	Retrospective case series	374 (17 elective)
Rimsans et al^25^	2018	Four-factor prothrombin complex concentrate for warfarin reversal in patients with left ventricular assist devices	Level III	Retrospective cohort study	37
Yang and Liu^26^	2018	Sequential cataract surgeries in a patient with a left ventricular assist device (LVAD)	Level V	Case report	1
Chen et al^21^	2017	Noncardiac surgery in patients with continuous-flow left ventricular assist devices: A single institutional experience	Level IV	Retrospective case series	209
Yoon et al^27^	2016	Pan-cardiac cycle fixed mitral valve opening in an LVAD patient presenting with hemorrhagic shock	Level V	Case report	1
Davis et al^3^	2015	Systematic review of outcomes after noncardiac surgery in patients with implanted left ventricular assist devices	Level II	Systematic review	161
Arnaoutakis et al^28^	2014	General and acute care surgical procedures in patients with left ventricular assist devices	Level IV	Retrospective case series	173
Ahmed et al^29^	2012	Elective noncardiac surgery in patients with left ventricular assist devices	Level IV	Retrospective case series	6
Morgan et al^12^	2012	Noncardiac surgery in patients on long-term left ventricular assist device support	Level IV	Retrospective case series	86
Kartha et al^30^	2008	Laparoscopic cholecystectomy in a patient with an implantable left ventricular assist device	Level V	Case report	1
Schmid et al^31^	2001	Noncardiac surgery in patients with left ventricular assist devices	Level IV	Retrospective case series	14
Goldstein et al^13^	1995	Noncardiac surgery in long-term implantable left ventricular assist-device recipients	Level IV	Retrospective case series	8

## Discussion

As the number of patients with LVADs presenting for elective surgeries increases, it is important that clinicians understand best practices in perioperative management to limit the incidence of complications. Improved generations of LVADs, complemented by further developed perioperative protocols, may provide beneficial outcomes for these patients.^14-16^ Our study presents a case series of 5 patients with LVADs who underwent 6 TJR procedures including 1 TSA, which, to our knowledge, has not been previously described in the literature. All patients demonstrated favorable outcomes with no reoperations. Complications addressed in the early perioperative period included transfusion in 2 patients, 1 readmission for GI bleeding shortly after the procedure, and 1 transient hypoxic episode that occurred shortly after surgery and responded to diuresis. Additionally, 4 patients were still living up to 20 months after surgery, which reinforces the potential longevity of patients with modern LVADs.

Limitations of this study include a single-center case series design, which may limit generalizability to other centers. Our institution is a quaternary referral center for heart failure with a large practice of heart transplant and advanced heart failure patients and so these results may not generalize to less experienced centers.

Our reported experience with 6 TJR procedures suggests that elective TJR may be safely performed with the application of appropriate perioperative protocols. Comparing findings from our case series to Rosenberg et al’s 2022 retrospective series (reported to be one of the largest published TJR-LVAD cohorts) reveals differences. Rosenberg et al provided up to 10 years of follow-up information on patients with LVADs (devices not specified) who underwent 12 total surgeries: 5 TKAs, 3 revision TKAs, 2 THAs, and 2 revision THAs.^8^ While Rosenberg et al’s cohort reported a 100% incidence of bleeding/thrombotic event, we reported only a 33% rate of bleeding event (2/6 surgeries) and no thrombotic complications. Moreover, the Rosenberg et al study reports a 40% mortality in revision TKA patients, whereas our case series observed no stroke-related mortality; the single mortality in our cohort occurred because of end-stage LVAD failure. Improvements in patient outcomes are likely attributed to modern LVAD technology, preoperative VAD optimization, and anticoagulation practices. In our study, the patient with the most complications had a HeartMate II device, which is older and may be more prone to complications than the HeartMate 3. While we are unable to draw definitive conclusions, it is possible that compared to older LVADs, the newer devices may lower complication rates; this should be examined in more detail as these patients undergo more elective procedures. Similar to the TKA case report by Sheikh et al,^7^ our single TKA patient experienced no complications postoperatively, although we used an adductor canal block as opposed to the neuraxial technique used in their study. The observed decreased burden of transfusion, lack of device thrombosis, and absence of infection-related reoperations across our patients suggest that modern LVAD technology and perioperative strategies may lessen potential risks of TJR within the long-term LVAD cohort. GI bleeding is a commonly observed morbidity across patients with LVAD implantation in prior studies of elective surgeries in this patient population, encompassed by risk factors including pre-LVAD GI bleeding, antiplatelet, and vitamin K antagonist use, right ventricular dysfunction, and a post-LVAD ejection fraction >30%, and is often managed by endoscopy.^17-20^

Our systematic review identified publications examining noncardiac elective surgeries in the LVAD population (Table 3). Many of these studies had a combination of emergent and elective surgeries and focused mostly on general surgery procedures (Supplemental Table 1). In one of the largest studies, Mentias et al identified Medicare patients with LVADs who underwent noncardiac elective and emergent surgeries.^11^ Of the 1326 patients in this category, the researchers found that 199 (15%) of these were orthopedic surgeries, with 27 patients undergoing elective TJR.^11^ They found a 30% 30-day readmission rate in elective surgeries including a 25% incidence of acute kidney injury, a 7% risk of major adverse cardiac event, and a 25% risk of blood transfusion. Taken together, these studies found rates of clinically significant bleeding or transfusion after elective surgery from 17% to 58%, while 30-day mortality rates and infection rates were reported at 4.3% in large national registries and 8.6% in single-center series, respectively.^11,13,21^ A 2025 study by Stevenson et al reported 1 patient developed GI bleeding with a prior history of the condition, similar to the THA patient observed in our case series.^22^ Notably, most existing studies detailed in our systematic review were completed years prior to current enhanced recovery protocols. Additionally, advancements in LVAD technology, particularly the transition from HeartMate II to HeartMate 3 have led to significantly lower pump thrombosis, stroke, and major bleeding.^14-16^

In conclusion, the results of this retrospective, single-institution case series and systematic review suggest that TJR may be considered in patients with modern LVADs, provided that robust perioperative management and multidisciplinary support are in place. While anticoagulation and bleeding risk remain important considerations, improvements in device design and perioperative management have substantially mitigated these concerns. To avoid complications, a coordinated, multidisciplinary approach with patient selection, standardized perioperative optimization, and communication is required.

## Supplemental Material

sj-docx-1-hss-10.1177_15563316261464135 – Supplemental material for Elective Total Joint Replacement in Patients With Left Ventricular Assist Devices: A Modern Case Series and Systematic Review Supplemental material, sj-docx-1-hss-10.1177_15563316261464135 for Elective Total Joint Replacement in Patients With Left Ventricular Assist Devices: A Modern Case Series and Systematic Review by Ramana Kolady, Natasha Singh, Heather L. Lander and Benjamin F. Ricciardi in HSS Journal®

sj-docx-2-hss-10.1177_15563316261464135 – Supplemental material for Elective Total Joint Replacement in Patients With Left Ventricular Assist Devices: A Modern Case Series and Systematic Review Supplemental material, sj-docx-2-hss-10.1177_15563316261464135 for Elective Total Joint Replacement in Patients With Left Ventricular Assist Devices: A Modern Case Series and Systematic Review by Ramana Kolady, Natasha Singh, Heather L. Lander and Benjamin F. Ricciardi in HSS Journal®

sj-docx-3-hss-10.1177_15563316261464135 – Supplemental material for Elective Total Joint Replacement in Patients With Left Ventricular Assist Devices: A Modern Case Series and Systematic Review Supplemental material, sj-docx-3-hss-10.1177_15563316261464135 for Elective Total Joint Replacement in Patients With Left Ventricular Assist Devices: A Modern Case Series and Systematic Review by Ramana Kolady, Natasha Singh, Heather L. Lander and Benjamin F. Ricciardi in HSS Journal®

sj-docx-4-hss-10.1177_15563316261464135 – Supplemental material for Elective Total Joint Replacement in Patients With Left Ventricular Assist Devices: A Modern Case Series and Systematic Review Supplemental material, sj-docx-4-hss-10.1177_15563316261464135 for Elective Total Joint Replacement in Patients With Left Ventricular Assist Devices: A Modern Case Series and Systematic Review by Ramana Kolady, Natasha Singh, Heather L. Lander and Benjamin F. Ricciardi in HSS Journal®

sj-docx-5-hss-10.1177_15563316261464135 – Supplemental material for Elective Total Joint Replacement in Patients With Left Ventricular Assist Devices: A Modern Case Series and Systematic Review Supplemental material, sj-docx-5-hss-10.1177_15563316261464135 for Elective Total Joint Replacement in Patients With Left Ventricular Assist Devices: A Modern Case Series and Systematic Review by Ramana Kolady, Natasha Singh, Heather L. Lander and Benjamin F. Ricciardi in HSS Journal®
