# Supplementary material for: Elective Total Joint Replacement in Patients With Left Ventricular Assist Devices: A Modern Case Series and Systematic Review
Source: HSS J. 2026 Jul 6:15563316261464135. Online ahead of print. doi: 10.1177/15563316261464135 (PMC13337564; doi:10.1177/15563316261464135)
Supplement: sj-docx-5-hss-10.1177_15563316261464135 – Supplemental material for Elective Total Joint Replacement in Patients With Left Ventricular Assist Devices: A Modern Case Series and Systematic Review [file sj-docx-5-hss-10.1177_15563316261464135.docx]

*Supplemental Table 1: Discussion of Included Studies on LVAD and Elective Surgery and Arthroplasty*

| **Citation** | **Elective Surgeries** | **Duration of Follow up** | **Elective Complications** | **Reoperations** |
| --- | --- | --- | --- | --- |
| Sheikh et al | 1 TKA | 6 months | None | None |
| Rosenberg et al | 12: 5 TKAs, 3 TKAs, 2 THAs, 2 revision THAs | Up to 10 years (individual follow up periods not reported, EHR from 2012 to publication) | All patients suffered bleeding/thrombotic event, 8/12 (66.7%) required intraoperative transfusion, 1/5 revision patients (20%) suffered LVAD thrombus. 2/5 (40%) patients passed away secondary to hemorrhagic stroke 22 and 32 months after LVAD implantation, or 16 months and 20 months after primary arthroplasty. | 1 (8.3%) patient had 2 infections - resulted in a 2-stage revision and debridement, antibiotic, and implant retention (DAIR) months later for subsequent infection. 1 patient (8.3%) underwent 2 stage revision arthroplasties for metallosis and concomitant infection. |
| Stevenson et al | 4 emergent, 13 elective: 5 esophagogastroduodenoscopies, 5 colonoscopies, 1 transurethral resection of prostate, 1 splenic artery embolization, 1 tooth extraction | Within 30 days of PCC4 administration | GI bleed (MCS-ARC type 3a) in patient with prior GI bleeding history (5.9%) – *not explicitly specified if in elective procedure or not* | None |
| Yahav-Shafir et al | 6 emergent, 17 elective (not specified which of 23 are elective): gastroscopy and colonoscopy, active bleeding, craniectomy, hematoma execution, tracheal stenosis resection and reconstruction, laparoscopic gastrostomy, below knee amputation, above knee amputation, thrombectomy lower limb, thoracotomy, hemothorax, transnasal polypectomy, laparoscopic appendectomy, gastroscopy, polypectomy, atrial flutter ablation, hemiarthroplasty, hip, hysteroscopy, cervical polypectomy, open inguinal hernia repair, diagnostic laparoscopy, small bowel obstruction, anal fistulotomy, tracheal stenosis resection and reconstruction, wide local excision with skin graft, ureteroscopy, lithotripsy, atrial flutter ablation, laparoscopic inguinal hernia repair | Up to 1-month post-surgery | 2 (8.7%) passed away, attributed to “non-anesthetic surgical interventions and multi-organ failure in ICU”. 9 (39.1%) patients received PRBC, 4 (17.4%) received FFP, and 1 received platelets. 4 patients (17.4%) not extubated post-procedure (“various clinical reasons”), 2 (8.7%) with tracheostomy. | 1 (4.3%) reoperation due to bleeding |
| Mentias et al | 1000 emergent/urgent, 326 elective: 155 general surgeries, 25 thoracic surgeries, 62 orthopedic surgeries, 48 genitourinary surgeries, 11 head and neck surgeries, 26 vascular surgeries | Not explicitly stated, mortality followed at least 60 days or more | 14 (4.3%) faced 30-day mortality, 82 (25.2%) Acute kidney injuries, 18 (5.5%) Sepsis, 27 (8.3%) Acute heart failures, 78 (23.9%) Blood transfusions, 23 (7.1%) Major adverse cardiovascular events, | *Not stated explicitly if reoperation,* 106 (32.5%) 30-day readmission |
| Zilbermints et al | 5 emergent, 5 elective: 1 bariatric surgery for morbid obesity, 1 hiatal hernia repair, 2 cholecystectomies, 1 small bowel resection for carcinoid tumor | Not explicitly stated | 1 elective sleeve gastrectomy patient and one elective cholecystectomy patient (40%) required postoperative blood transfusions | None |
| Vigneswaran et al | 17: 7 enteral access placements, 6 cholecystectomies, 2 hernia repairs, 1 small bowel resection and 1 splenectomy | Not explicitly stated but likely 90 days (reported no 90-day mortalities) | 5/17 (29.4%) required intraoperative blood transfusion. In immediate postoperative period, 7 (41.2%) patients had anemia. 1 (5.9%) groin hematoma after inguinal hernia repair. 1 (5.9%) hematoma after bilateral inguinal hernia repair. 1 (5.9%) 30-day mortality in patient with gastronomy food tube. | None |
| Rimsans et al | 30 urgent/emergent, 19 elective: 3 biopsies,  5 cardiac catheterizations, 5 screening endoscopies/colonoscopies, 1 hernia repair, 1 amputation of toes, 2 thoracentesis, 1 peripherally inserted central catheter (picc) placement, 1 laparoscopic sleeve gastrectomy | 30 days | No explicit distinction between elective and urgent outcomes, no complications | None |
| Yang, Liu | 2 (cataract) | 16 months | None | None |
| Chen et al | 43 urgent, 35 elective | Not explicitly mentioned, states median survival after LVAD implant is reported as 1737 days | 3 infections (8.6%), 6 bleeding (17.1%), 2 wound breakdown (5.7%), 3 other: transient episode of aphasia and confusion after carotid artery stenting, air leak after lobectomy, and a bowel obstruction after hysterectomy (8.6%) | None |
| Yoon et al | 1 laparoscopic cholecystectomy | 12 months | Hemorrhagic Shock (POD 9) | None |
| Davis et al | 54 (Bhat), 6 (Ahmed), 22 (Morgan), 1 (Goldstein) | 30 days | Morgan - Reported 9/25 bled (36%) | None |
| Arnoutakis et al | 21 emergency, 8 urgent, 38 elective (not specified which were elective) | Intraoperative mortality and 30-day mortality recorded. Primary endpoints of study were heart transplantation or death. | Unable to determine | None |
| Ahmed et al | 6 :1 radical prostatectomy, 1 femoral endarterectomy, 1 arthroscopic rotator cuff repair, 1 laparoscopic sleeve gastrectomy, 1 laparoscopic hemicolectomy, 1 exploratory laparoscopy | 2075 days for radical prostatectomy, 575 days for femoral endarterectomy, 441 days for arthroscopic rotator cuff repair, 586 days for laparoscopic sleeve gastrectomy, 871 days for laparoscopic hemicolectomy, 30 days for exploratory laparoscopy | 1 patient (16.7%) who underwent laparoscopic hemicolectomy died of heart failure complication 2.4 years after surgery | None |
| Morgan et al | 3 emergent, 22 elective: 4 inguinal hernia repair, 4 cholecystectomy, 3 bilateral salpingo-oophorectomy, 2 colon resection, 2 excision of lipoma, 2 insertion of catheter, 1 endometrial ablation, 1 iliofemoral bypass, 1 gastric bypass, 1 teeth extraction, 1 removal of catheter | Not explicitly stated, but non-cardiac surgeries (NCS) were performed at a median duration of 285 days on left ventricular assist device (LVAD) support. Study also reports midterm survival, noting no significant difference in 1-year survival between LVAD patients who underwent NCS and those who did not, suggesting follow-up data collected at least 1-year post-LVAD implantation. | Bleeding requiring transfusion of packed red blood cells (PRBCs), occurred in 9 of 25 procedures (36%). *Not stated if all complications were for elective, but majority of procedures were elective* | 3 (13.6%) due to bleeding complications requiring transfusion: Inguinal hernia repair, Cholecystectomy, Colon resection |
| Kartha et al | 1 laparoscopic cholecystectomy | 2 days | None | None |
| Schmid et al | 20 emergent, 1 (not emergent/urgent, presumed elective): pleural decortication | 94 days | 1 (100%) death at 94 days following procedure (states total LVAD support duration of 324 days, and procedure took place 230 days after LVAD implantation) with late outcome of Multiple Organ Failure (MOF) | None |
| Goldstein et al | 12 (presumed all elective): 2 tracheostomies, left pleural decortication, sternal debridement and pectoralis muscle flap, lvad pocket debridement and rectus abdominis muscle flap, plication of bleeding gastric ulcer, nephrectomy, right lower lobectomy, groshang catheter insertion, sternal debridement, multiple tooth extraction, debridement sacral decubitus ulcer and local flap | Not explicitly stated | 2 patients (16.7%) succumbed to sepsis unrelated to surgical procedure, 1 patient (8.3%) died after driveline rupture. 7 patient complications (58.3%) Left pleural decortication – Hypotensive with positional change, LVAD pocket debridement and rectus abdominis muscle flap - bleeding, plication of bleeding gastric ulcer - hypotension, nephrectomy -hypotensive with positional change, right lower lobectomy - hypotensive with positional change, sternal debridement - reintubation for dyspnea, debridement sacral decubitus ulcer and local flap -hypotensive with positional change | None |

.
